# The Effects of Earthquake Experience on Disaster Education for Children and Teens

**DOI:** 10.3390/ijerph17155347

**Published:** 2020-07-24

**Authors:** Da-Hye Yeon, Ji-Bum Chung, Dong-Hyeon Im

**Affiliations:** School of Urban and Environmental Engineering, Ulsan National Institute of Science and Technology, Ulsan 44919, Korea; yeondahye@unist.ac.kr (D.-H.Y.); donghyune@unist.ac.kr (D.-H.I.)

**Keywords:** disaster education, cognitive response, emotional response, educational effectiveness

## Abstract

The purpose of this study is to examine the factors of disaster experience that impact the effectiveness of disaster education on school students (children and teens). Following the magnitude 5.4 Pohang earthquake in 2017, Pohang City Hall conducted a school earthquake disaster education program over a period of four months (August to November) in 2018. Professors and graduate students from the Ulsan National Institute of Science and Technology taught around 5000 middle and high school students, while also conducting surveys. The experiences of the Pohang earthquake were analyzed and divided into cognitive responses and emotional responses. Students who felt activated emotional responses, surprise and fear, but not joy, tended to have more effective educational experiences. On the other hand, unpleasant emotional reactions, such as anger and sadness, had a negative effect on educational effectiveness. The cognitive response, which is perceived intensity in this research, did not impact educational effectiveness significantly. These results imply that the emotional responses of students are more important than their cognitive responses in providing a disaster education program. This means that even though an earthquake may be small in magnitude and may not cause physical damage, we still need to provide immediate disaster education to the children and teens if they are surprised and afraid of future disasters.

## 1. Introduction

**Figure 1:**
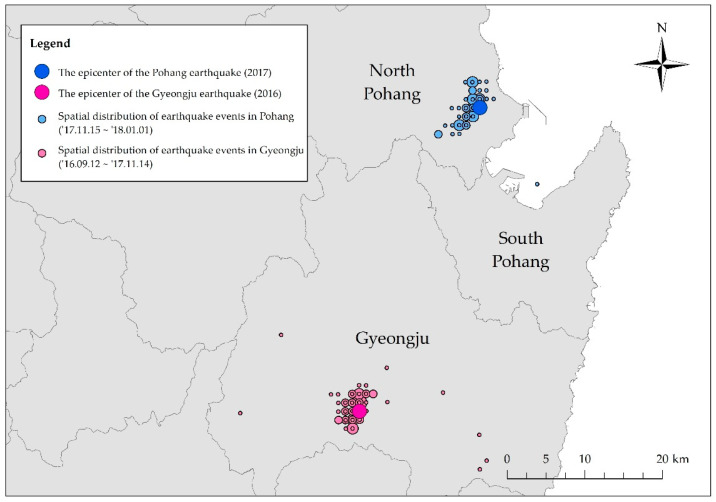
Location of the epicenters of the Pohang and Gyeongju earthquakes, and the spatial distribution of seismic events near the epicenters.

The frequency of earthquakes in Korea has historically been low, and their size has been relatively small. Therefore, up until 2016, the contents of Korea’s disaster education program did not include how to respond to earthquakes, because people viewed the country as being safe from such incidents. However, two seismic, unprecedented events then took place in Korea. In 2016, an earthquake of Magnitude 5.8 (M 5.8) occurred in Gyeongju, which was followed by the Pohang earthquake of M 5.4 about one year later, on 15 November 2017 (see Table 1). The distance between the epicenter of these two earthquakes was about 40 km. Figure 1 shows the location of seismic events in Pohang and Gyeongju. Although the magnitude of the Gyeongju earthquake was larger than the one in Pohang, the damage inflicted on Pohang was greater [1].

The seismic waves of the Pohang earthquake shocked the entire population of Korea, causing the Korean government to postpone the National College Scholastic Aptitude Test, which had been scheduled for November 16. Following this decision, Korean schools felt the need to offer earthquake response education to students, and the first steps toward offering earthquake disaster education were put in place [2]. For example, Pohang City Hall planned a disaster education program designed to ensure the safety of students by training them protective action for when an earthquake occurs.

The effects of disasters can be substantially reduced if people are well-informed about how to respond to them. Disaster education plays an important role in improving students’ disaster awareness and preparedness level [3]. Furthermore, as the Sendai Framework for Disaster Risk Reduction [4] stated that children and youth can be the “agents of change” in transforming and improving the safety of society. The participants of disaster education programs tend to be associated with realistic risk perceptions, increased disaster mitigation knowledge, and increased discussions among family members about disasters [5].

In the Republic of Korea, schools and public institutions have provided many different forms of disaster educations. They offer knowledge of hazards, causes of injury, protective actions, and mitigation actions, and these activities can help people improve resilience, survival, and mental trauma recovery. Despite these efforts, many children and teens still either do not know how to respond to disasters [6] or fail to take any preparatory measures [7]. However, there have not been many studies that have evaluated disaster education programs or their effectiveness [8]. In particular, it is difficult to find research evaluating the effects of disaster experiences on disaster education.

Having disaster experience is one of the key factors affecting an individual’s disaster preparedness behaviors, as people who have experienced disasters either directly or indirectly face different levels of intensity and damage compared to those who have not. In the case of children and teens, it is more important to investigate their emotional reactions to disaster experiences, as such feelings linger in their minds for a long time and affect their subsequent behavior [9]. Therefore, when determining the effectiveness of disaster education, we need to consider people’s disaster experiences and their emotions.

The purpose of this study is to analyze the earthquake experiences of children and teens and evaluate the effects of their experiences on disaster education. Educational effectiveness in this study will be measured using the students’ knowledge level regarding earthquake drills and their levels of training satisfaction.

## 2. Literature Review

### 2.1. Disaster Education for School Children and Teens

Experiencing a disaster when you have an immature psyche can be more stressful, leaning toward having a negative effect on a young person’s growth. Although the disaster may have occurred a long time ago, children can still remember such events vividly [10]. Accordingly, disaster education for children and teens is important to improve their resilience to disasters [11,12,13].

Various forms of disaster education were conducted: lectures [14,15,16], drills [17], discussions with teachers and friends [13,18], reading and listening [19], using hypermedia systems [20], games [21,22], after-school activities [12], and on-line intercultural projects [23].

Disaster education can be conducted across several levels. The individual level tends to improve the awareness of the disaster risk, and the community level, including home-based education, increases the adjustment and preparedness activities [13]. However, the most common and important type is school-based education [24,25]. Schools, as information communicators, can effectively enhance students’ safety knowledge, help with planning for a disaster situation, and structuralize mitigation [26].

### 2.2. Assessment of Disaster Education

To provide effective disaster education for children and teens, it is important to determine how the effectiveness of education can be measured. Usually, the measurement indices are devised by methodologies such as surveys, interviews, and observations. These indices include fear and risk perception [27,28], disaster risk knowledge, disaster risk reduction skills [3,17,18,29], attitude to disaster [23], confidence to respond against disasters [12], other preparedness activities [13,28], and student satisfaction [24].

In the case of applying new teaching methods, the methods itself were mainly used as factor [20,22]. Among all these factors, in general, students’ direct assessment of disaster prevention programs consists of learning satisfaction and effectiveness [30]. Johnson (2014) found that many studies used knowledge-based outcome indicators, such as “knowledge of hazard risks” and “knowledge of protective actions during an emergency” [8], and pointed out the necessity of developing an evaluation index.

However, there are not many studies about individual factors that can increase the effectiveness of disaster education, given the importance of individual attributes [19]. This may be a reason why many youths do not know what to do in an emergency, although institutions have offered disaster education [6].

### 2.3. Disaster Experience and Preparedness

Previous studies examined disaster experience as a factor that motivates people to prepare for future incidents [6,31]. More specifically, the physical, financial, and emotional damages inflicted by earthquakes, encourage people to prepare for the reoccurrence of such events [32]. The number of earthquakes experienced [33], the combination of the level of fear and the amount of damage suffered by homeowners [34], proximity to the earthquake epicenter, earthquake-related experiences, fear, and levels of pre-earthquake preparedness [32,33] are factors that have been identified for motivating preparedness.

Disaster experience is a major factor in promoting preparedness [35,36]. This means that victims who suffer from a disaster are better prepared to cope with future disasters. Furthermore, disaster experience also strengthens an individual’s ability to perceive disasters or risks, as well as their understanding of the actual situation [6,36]. Therefore, it is important to analyze disaster experience and examine its impact on preparedness.

However, such an experience can also have negative effects on levels of preparedness. For example, experiencing a moderate disaster or less traumatic exposure can cause “a false sense of confidence” or normalcy bias [35,37]. When the disaster is not catastrophic, people may go through minor seismic experiences and eventually consider themselves to be safe from potential earthquake risks. This may result in people being reluctant to engage in disaster preparedness activities in advance [38].

Several attempts have been made to interpret or define disaster experiences, which can be measured using their intensities and the level of physical, emotional, and financial damage inflicted [32]. Becker et al. focused on the emotional experiences, which were classified into the following categories: direct experience, indirect experience, vicarious experience, and life experience [35].

In this study, the earthquake experiences of children and teens were analyzed using both their emotional and cognitive response. The cognitive response factor includes the participants’ perceived Modified Mercalli Intensity Scale degree. The measurement of the Modified Mercalli Intensity (MMI scale) is a mechanism of intelligent judgement and observation that can be determined by the observation of each case, such as the building or geologic condition [39]. In contrast, an individual’s emotional response can be independent of their cognitive understanding [34,40], and is more powerful than a cognitive judgment [41]. For example, the emotional response following flooding encourages people to prepare more thoroughly [34].

### 2.4. Disaster and Emotions

In disaster situations, negative emotions constitute the principal response to risks and threats [9]. However, people do not experience purely negative emotions in disaster situations, and their reactions may depend on both the scale of the damage they faced and personal characteristics. In particular, each age group has a different reaction to a disaster, and we need to pay greater attention to children due to how non-typical their reactions are [42]. Therefore, it is necessary to comprehensively examine children and teens’ emotional reactions to disaster experiences [43].

The common emotional reactions to a disaster are fear, anger, guilt, and sadness [42]. To understand emotions comprehensively, previous research has studied the classification of emotions. Ekman et al. (1980) categorized emotional experiences through facial expressions in order to develop a classification system that is simply divided into positive or negative. In his paper, the primary emotions are disgust, surprise, sadness, fear, pain, and arousal [44]. Shaver et al. (1987), meanwhile, suggest that the basic emotions are fear, joy, surprise, sadness, anger, and love [45], while Parrott (2001) classified these emotions as love, joy, surprise, anger, sadness, and fear [46]. Russell (1980) proposed the “Circumplex Model.” This model has two dimensions: the degree of activation and the degree of pleasure. For example, surprise and fear are activated emotions, while anger, disgust, and sadness constitute unpleasant emotions [47].

Emotions represent the strongest system for motivating an individual’s behavior [48], with each emotion triggering a specific behavior or tendency [49]. Fear and worry tend to induce evacuation and preparedness to avoid danger, while joy is related to encouraging advances and protecting a person’s life. Anger makes people want to destroy or punish the cause, while sadness has a propensity to make people give up their willingness to act and seek comfort by accepting their loss [9]. Emotion in particular can affect an individual’s motivation for learning preparedness and survival skills. Arousing fears of health risks has a positive impact on the attitude toward acceptance of recommendations. People who are in a high state of fear take the health recommendations more seriously and intend on following it [50]. In neuroscience, the learning ability of those who have fearful faces are better than those who have neutral faces [51].

## 3. The School Disaster Educational Program for Children and Teens in Pohang City

**Figure 2:**
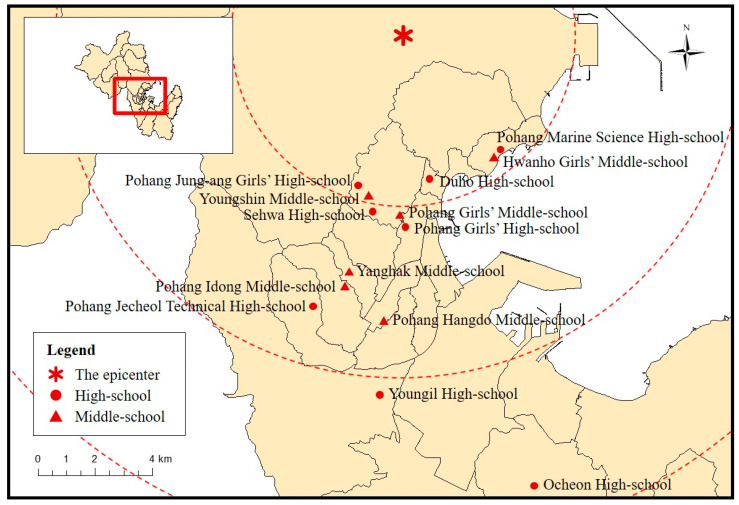
Location of the Epicenter (asterisk) and Schools Participating in the Educational Program (circle and triangle).

In 2018, Pohang City Hall’s “Earthquake Damage Settlement Association” and “Disaster Prevention Policy” department established a joint plan to provide a customized earthquake disaster educational program for children and teens. The purpose of the program, which was based on the earthquake response manuals of the Korean government, was to ensure the safety of school students from future earthquakes. As part of the plan, the “2018 school earthquake disaster education program in Pohang” was conducted over a period of four months (August to November) in 2018. The content was taught by professors and graduate students from the Ulsan National Institute of Science and Technology (UNIST), who also conducted surveys regarding the program’s effectiveness. Fourteen schools (six middle and eight high schools) in Pohang city participated, and the number of students who joined the program was about 5000. Figure 2 shows the schools that participated in the program. All of the schools are located within 24 km of the epicenter of the Pohang earthquake in November 2017. 

The training content consisted of three parts. The first part focused on earthquake science, including the phenomena of earthquakes and seismic waves. When describing seismic waves, students were taught the difference between P waves and S waves using experiments and video materials. The second part concerned earthquake-resistant and earthquake-proof construction, with key elements and goals regarding the seismic design explained. In the final section, the general protective action and strategy regarding earthquakes was introduced, as well as memorization skills designed to help students remember key actions easily.

## 4. Methods

### 4.1. Research Design

This study examined factors relating to the disaster experience increase the effectiveness of earthquake response education for the students who experienced the Pohang earthquake. The experience was measured by two responses to the earthquake: cognitive response and emotional response. The cognitive response was determined by the perceived scale of the earthquake. The emotional response was measured by five emotions: fear, joy, surprise, sadness, and anger. The variables for the effectiveness of education were measured by the knowledge level of the earthquake protection drills and the level of satisfaction of the education program. The structural equation model (SEM) was conducted to find the statistically significant factors from students’ previous disaster experience that impacted educational effectiveness.

**Figure 3:**
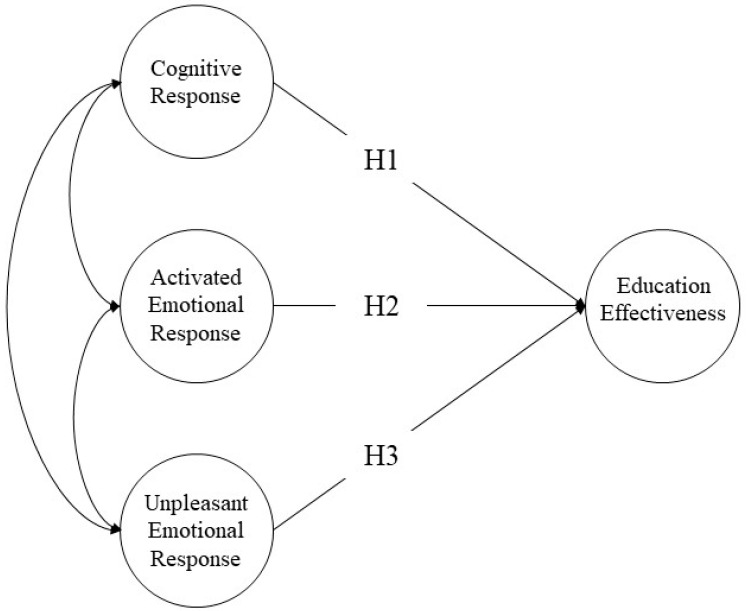
Structural Equation Model for Educational Effectiveness with Hypothesis (H).

The hypothesis of this study is that cognitive response and two emotional responses (using the activated emotional and unpleasant emotional response categories developed by Russell [47]), stemming from students’ previous disaster experiences, will have an impact on educational effectiveness. Figure 3 shows the hypotheses of this paper.

**Hypothesis 1** **(H1).** *Cognitive response will positively influence educational effectiveness*.

**Hypothesis 2** **(H2).** *Activated emotional response will positively influence educational effectiveness*.

**Hypothesis 3** **(H3).** *Unpleasant emotional reaction will negatively influence educational effectiveness*.

Two responses to the earthquake were used both to analyze the previous disaster experience and identify the factors influencing educational effectiveness. The latent endogenous variable was educational effectiveness, which was measured through a combination of knowledge level and level of satisfaction. The latent exogenous variables were cognitive response, activated emotional response, and unpleasant emotional response. The observed exogenous variables for emotional response included five emotions (fear, joy, surprise, sadness, and anger), which were classified into two latent variables. The activated emotional response was measured using fear, joy, and surprise, while the unpleasant emotional response was measured using sadness and anger.

### 4.2. Data Collection

The survey data were collected as a part of the “2018 school earthquake disaster education program in Pohang” developed by Pohang City Hall. The survey was conducted from August to November (four months) in 2018, about a year after the Pohang earthquake. The respondents were about 5000 students from six middle schools and eight high schools in Pohang. Prior to the survey, the earthquake response education was conducted by members of UNIST. The data of 3316 respondents were analyzed after eliminating insincere answers and missing values from among the initial ~5000 questionnaires. Table 2 shows the demographic information of the target respondents. In both middle and high schools, the proportion of women was higher. The questionnaire has five parts: location when the earthquake occurred, perceived scale, emotional response, training assessment, and demographic characteristics.

### 4.3. Survey Questionnaire

#### 4.3.1. Earthquake Intensity

The question designed to gauge students’ cognitive response concerned how they perceived or observed their surroundings and intensity during the shock (“When the earthquake occurred, what happened around you? Choose one.”). The statement about magnitude was based on the Modified Mercalli Intensity scale, developed by Harry O. Wood and Frank Neumann. Several additional features were also included in the survey, such as the middle size (**M**5.4) earthquake and the level of damage. The scales are “1 = Not felt,” “2 = Felt only by a few persons at rest,” “3 = Felt quite noticeably. Windows and furniture shook,” “4 = Flowerpots or furniture fell down or windows shook violently,” “5 = Some windows broken or walls cracked,” “6 = Walls or buildings collapsed” [39].

#### 4.3.2. Emotional Response

The questions concerned five different emotions: fear, joy, surprise, sadness, and anger (“When you experienced an earthquake at first, you could feel various emotions. How did you feel about the earthquake in Pohang?”). These items were measured using a five-point Likert-type scale (“1 = not at all,” “2 = hardly,” “3 = somewhat,” “4 = fairly,” and “5 = very.”).

#### 4.3.3. Knowledge Level

The respondents answered six right or wrong questions concerning earthquake response actions. To measure the educational effectiveness, the six questions were based on the content of the education program (“Here are six yes or no questions about earthquake response actions. If the statement is right, you should choose yes. Check your answer”). The six questions were as follows: (1) “If you are indoors, right away, you must try to get under a desk or table to protect your head and torso” (correct answer: yes); (2) “During the shaking, you must evacuate outside of buildings immediately” (no); (3) “During the shaking, you must use an elevator to evacuate the building as fast as possible” (no); (4) “When the earthquake occurs, you must evacuate to open space, such as a park or a ground” (yes); (5) “If you are driving, you must use a vehicle to move to a safe place” (no); and (6) “If you are outside, you must stay next to the walls” (no). The questions and their contexts are based on the manual of the Ministry of the Interior and Safety.

#### 4.3.4. Level of Satisfaction

The level of educational effectiveness was assessed by the students. They answered questions regarding their level of satisfaction with the training (“I am satisfied with this earthquake response training.”) using a five-point Likert-type scale (“1 = strongly disagree,” “2 = disagree,” “3 = neither agree nor disagree,” “4 = agree,” and “5 = strongly agree.”).

### 4.4. Data Analysis

The analysis was conducted using the IBM SPSS Statistics (IBM, Armonk, NY, USA) and The R Project for Statistical Computing (The R Foundation for Statistical Computing, Vienna, Austria). The basic statistical characteristics and correlations among the variables were found through frequency analysis, correlation analysis, and regression analysis. The Structural Equation Model was applied to evaluate the causal relationship between earthquake experience and training effectiveness. The five emotional response variables were converted into two factors through Principal Component Analysis (PCA). The internal consistency, Construct Reliability, and Average Variance Extracted were then determined to confirm the validity of the PCA results.

## 5. Results

### 5.1. Response to Past Eartqhuake Experiences

**Figure 4:**
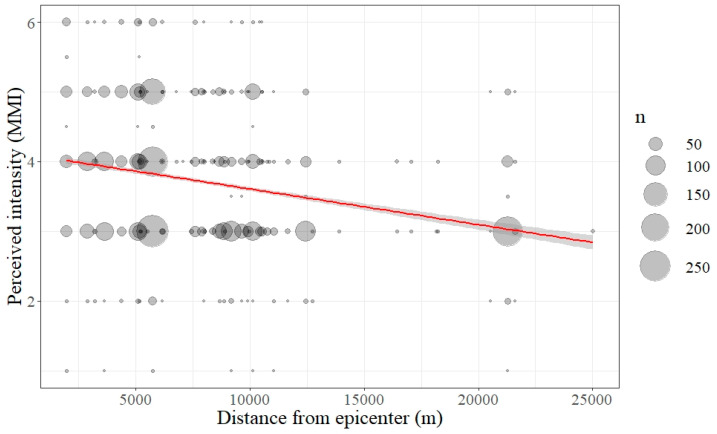
Level of Perceived Earthquake Intensity (Modified Mercalli Intensity (MMI)) and Distance from the Epicenter. The red line represents the smoothing trend line, and the gray belt around red line shows standard error. The perceived intensity decreases with distance from the epicenter.

All of the respondents to this survey had experienced the Pohang earthquake. The result shows that the respondents perceived an intensity of 3.7 MMI (Mean (M) = 3.71, Standard Deviation (SD) = 0.89) on average. The perceived MMI tended to decrease as the distance from the epicenter increases (see Figure 4). When compared by gender, female students (M = 3.75, SD = 0.91) tended to recognize a stronger scale than male students (M = 3.61, SD = 0.85), and there is a statistically significant difference between male and female students (t-test = −4.166, *p* = *0*.000). According to the ANOVA test results, there were no significant differences between male high vs. male middle school students (*p* = *0*.807). The same is also true of female high vs. female middle school students (*p* = *0*.912).

In contrast, the survey results show that the strongest emotional response during the Pohang earthquake was surprise (M = 4.33, SD = 0.89), while the weakest emotion was anger (M = 2.00, SD = 1.11). The mean values of the other emotions are fear (M = 3.91, SD = 1.09), sadness (M = 2.53, SD = 1.26), and joy (M = 2.17, SD = 1.18). According to the result of the independent group t-test between emotional responses and gender, there are significant differences (*p*-value = 0.000), and joy and other emotions show an opposite tendency. While most emotional responses are more strongly experienced by females, males rated joy higher than females. The correlation between respondents’ emotional responses and proximity to the epicenter is significant. The nearer the location of the respondent, the stronger the level of emotion other than joy. By contrast, the correlation coefficient between the level of joy and distance is positive (corr. = 0.096, *p* < 0.01). Age has a statistically significant relationship with joy (corr. = −0.042, *p* < 0.05) and anger (corr. = 0.082, *p* < 0.01).

The Pohang earthquake was not as big an earthquake compared with other significant examples, and some students thought it was an interesting experience. As joy is not a common response during a disaster, this shows unique characteristics that differ from other emotions. More specifically, students who were male, younger, and far away from the epicenter felt higher levels of joy. The other reactions, including anger, had contrasting tendencies. These results show that the emotional response to disasters varied according to age, gender, and location during the event.

Exploratory factor analysis was used to reduce the dimensions of the five emotional responses and examine the classification hypothesis (see Table 3). It analyzed two latent factors: activated emotion and unpleasant emotion. The five emotional responses were reduced to two components by applying principal component analysis (PCA). The activated emotional factor includes fear, joy, and surprise, while the unpleasant emotional factor covers sadness and anger. The reliability analyses used Cronbach’s alpha and AVE to validate the reliability of the model. The internal consistency (Cronbach’s α) of each factor was 0.692 and 0.693. The AVE value of each factor was 0.544 and 0.771.

### 5.2. Effect of Disaster Education

Respondents completed six questionnaires in order to evaluate their level of satisfaction regarding the training. The quizzes consisted of yes/no type questions. For each problem, one point was given for the correct answer and zero for the wrong answer, meaning that the lowest score is zero and the highest score is six. The average score of respondents was 5.16 (SD = 1.04) out of 6.00. Satisfaction levels regarding the earthquake response training were measured using a five-point Likert-type scale, from “1 = not at all” to “5 = strongly agree.” The mean value was 4.61 (SD = 0.71) out of 5.00.

### 5.3. Factors Affecting the Level of Preparedness

**Figure 5:**
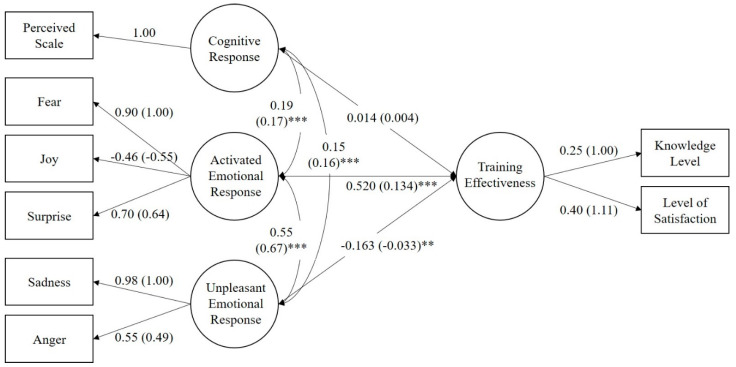
Causal Structural Equation Model (SEM) Diagram Showing Educational Effectiveness by Cognitive Response and Emotional Response with standardized path loading (estimate path loading). (** *p* < 0.01, *** *p* < 0.001).

The SEM (Structural Equation Model) was formed on the hypothesis that the factors relating to earthquake experience will enhance the effectiveness of the emergency training. Figure 5 shows the causal diagram for the model with an assessment of standardized (unstandardized) path loadings, β.

As we can see in Table 4, the model is reasonably consistent with the data, as the overall model fit indices are within the recommended values. The only exceptions to this are the indices $\chi^{2}$ significance and $\chi^{2}$/d.f. [52,53,54]. However, the $\chi^{2}$ significance and $\chi^{2}$/d.f. indices tend to increase in line with the sample size. As the sample size of this study is very large compared to typical samples (N = 200–300) of SEM, we will accept this model considering the overall significance of the test results across the entire model [54].

Table 5 shows the results of hypothesis testing. The standardized coefficient of H2 was 0.134 (*p* = 0.000), while for H3 it was −0.033 (*p* = 0.03). Therefore, we can accept hypotheses H2 and H3: namely, that the activated (unpleasant) emotional response will positively (negatively) influence educational effectiveness. However, the standardized estimate of H1 was only 0.004 (*p* = 0.721). As this was not a statistically significant result, we rejected the H1 hypothesis, which stated that the cognitive response (perceived MMI) would positively influence educational effectiveness. This result shows that an individual’s emotional response is more relevant to the effectiveness of disaster training than the recognized scale by witnessing or experiencing such incidents. More specifically, activated emotions (fear, surprise, and joy) have the greatest influence among the factors, while unpleasant emotions (anger and sadness) have a statistically significant but opposite influence. In other words, sadness and anger demotivate students to learn about the disaster.

## 6. Discussion

The experiences of the Pohang earthquake of M 5.4 in 2017 were analyzed and divided into cognitive responses and emotional responses. The cognitive responses of the students did not impact educational effectiveness significantly. This factor was measured using the earthquake intensity that the students perceived, which was measured to be around 3.7 MMI on average. The measured intensity result differed according to the individual’s proximity to the epicenter; students who were further away from the epicenter tended to experience weaker shaking. The reason why the effects of cognitive responses are not statistically significant may be due to the characteristics of the Pohang earthquake. Although its size was M 5.4, the second strongest event recorded in South Korea since 1978, it was only a mid- or minor-sized earthquake compared to the large magnitude earthquakes witnessed in Japan or other countries around the world. Consequently, in spite of the respondents’ surprise and fear, which were based on the unfamiliarity of experiencing earthquake, the physical damage inflicted by the earthquake was not very serious. Consequently, students might think that they do not need additional preparation because there was no notable damage.

Students felt various emotional reactions, including both negative and positive responses. The two emotional responses, activated emotional response and unpleasant emotional response, were statistically significant in terms of educational effectiveness (*p* < 0.01), with activated emotional responses identified as most significant factor. Students who felt surprise and fear, but not joy, tended to have more effective educational experiences. On the other hand, the unpleasant emotional reactions, such as anger and sadness, had a negative effect on educational effectiveness.

These results imply that students’ emotional responses are more important than their cognitive responses in terms of providing an effective disaster education program. This means that even though an earthquake may be small in terms of magnitude or physical damage caused, we still need to provide immediate disaster education to children and teens when they are surprised and afraid of future disasters. As the results of the analysis indicate, if children and teens are emotionally stimulated, their motivation to learn can be increased and can consequently increase the effectiveness of disaster education. Previous research supports the idea that “the fear-arousing communications” have a positive effect on behavioral change, such as recommendation acceptance [50]. Therefore, it is very important for school agencies to provide disaster education to the children and teens who are emotionally stimulated due to disasters. If these education programs are conducted right after a disaster has occurred, it would be more effective in successfully enhancing students’ knowledge of disasters and protective action, substantially contributing to reducing the damage caused by future disasters.

The emotional response, however, has both a positive and a negative impact on the effectiveness of disaster educations. If children and teens feel severe sadness and anger during a disaster, this can decrease the effectiveness of disaster education. In such cases, students feel helpless and tend to give up the willingness to learn helpful information designed to protect them from future disasters [9]. The most representative example of these negative emotional impacts can be found in some tragic cases relating to the loss of parents due to large disasters. In these cases, we need to provide post-traumatic stress disorder education, rather than the usual forms of disaster education.

## 7. Conclusions

The purpose of this study was to examine the factors relating to disaster experiences that impact the effectiveness of disaster education on school students (children and teens). As shown in the results, the “2018 school earthquake disaster education program in Pohang”, developed by Pohang City Hall, was meaningful. The M 5.4 Pohang earthquake in 2017 was a mid-sized earthquake compared with larger incidents, such as the M 8.0 Sichuan earthquake in China in 2008 or the M 9.0 Tohoku earthquake in Japan. However, the Pohang earthquake was the second largest earthquake in Korean history, and almost all people in Pohang were shocked by it because it was so unfamiliar. In particular, the surprise and fear of children and teens in Pohang city was severe. These activated emotional states motivated students in Pohang to learn about disaster science and how to take protective actions effectively. Although the education program started nine months after the earthquake, the vivid emotions of surprise and fear still lingered in the hearts of children and teens in Pohang city.

Educational institutions need to clearly understand the importance of students’ emotional conditions when planning a disaster education program. Furthermore, it is also important to change students’ perceptions regarding the controllability of the disaster. The message that preparedness can reduce the resulting damage may induce students to prepare more thoroughly [7], especially when the children and teens in question are emotionally stimulated.

## Figures and Tables

**Table 1:** The Pohang earthquake and the Gyeongju earthquake [1].

Earthquake	Date	Time (UTC *)	Location of epicenter		Depth (km)	Magnitude
			Latitude	Longitude		
Pohang earthquake	15 November 2017	05:29:31	36.11°	129.37°	7	5.4
Gyeongju earthquake	12 September 2016	11:32:54	35.76°	129.19°	15	5.8

* Universal Time Coordinated.

**Table 2:** The demographic information of subjects.

Total		Number	Percentage (%)
		*n* = 3316	100
Gender	Male	1053	31.76
	Female	2263	68.24
School	High school	1799	54.25
	Middle school	1517	45.75
The year of birth	1999	3	0.09
	2000	191	5.76
	2001	839	25.30
	2002	766	23.10
	2003	526	15.86
	2004	457	13.78
	2005	531	16.01
	2006	3	0.09

**Table 3:** Result of Principal Component Analysis of Emotional Response.

Emotional Response	Factor1	Factor2	Communality
Surprise	0.850	0.041	0.724
Fear	0.839	0.263	0.773
Joy	−0.622	−0.174	0.417
Anger	0.033	0.920	0.848
Sadness	0.400	0.769	0.750

**Table 4:** Model Fit Indices with Recommended Values (*n* = 3316).

Statistic	Recommended Value	Obtained Value
$\chi^{2}$	-	198.605
d.f.	-	15
$\chi^{2}$ significance	*p* > 0.05	0.000
$\chi^{2}$/d.f.	<5.0	13.240
RMR	<0.05	0.036
RMSEA	<0.08	0.061
TLI	>0.9	0.929
NFI	>0.9	0.959

**Table 5:** Hypothesis Testing Results and Unstandardized Path Loadings (*n* = 3316).

		Estimate	Standardized Error	*z*-Value	*p*-Value	Standardized Value
Hypothesis 1:	Perceived Earthquake Scale → Preparedness	0.004	0.011	0.357	0.721	0.014
Hypothesis 2:	Emotional Response 01 → Preparedness	0.134 ***	0.024	5.583	0.000	0.520
Hypothesis 3:	Emotional Response 02 → Preparedness	−0.033 **	0.011	−2.922	0.003	−0.163

** *p* < 0.01, *** *p* < 0.001.
